# Public libraries to promote public health and wellbeing: a cross-sectional study of community-dwelling adults

**DOI:** 10.1186/s12889-024-18535-5

**Published:** 2024-05-03

**Authors:** Manisha Karki, Marie Line El Asmar, Eva Riboli Sasco, Austen El-Osta

**Affiliations:** Self-Care Academic Research Unit (SCARU), Department of Primary Care & Public Health, Imperial College London, 323 Reynolds Building, Charing Cross Hospital, St Dunstan’s Road, London, W6 8RP UK

**Keywords:** Self-care, Libraries, Prevention, Community development, Public engagement, Health, Wellbeing, Loneliness, Library staff

## Abstract

**Background:**

Libraries in the UK have evolved from traditional book-lending institutions into dynamic community hubs, This study aims to explore the potential of libraries to act as community hubs to promote mental and physical health and wellbeing of community-dwelling adults, drawing on insights from both library users and library staff in England.

**Design:**

A mixed-method, cross-sectional study using online survey and interviews with community-dwelling adults and library staff.

**Methods:**

We collected data using a 14-item electronic survey and interviews with library users and staff to gauge perceptions. Descriptive statistics and thematic analysis were used to identify key trends and emergent themes.

**Results:**

We included 605 respondents from the survey and interviewed 12 library users and staff. Libraries remain popular and are considered a ‘safe place by members of the community, regardless of their frequency of service usage irrespective of whether they are frequent users of services. However, a lack of awareness among library users about community-facing services could act as a hurdle to improving community health and wellbeing. Targeted engagement with residents is needed to increase awareness of libraries’ services, including community interventions to help tackle loneliness and inequalities in digital and health literacy. Library staff often did not feel involved in important decision-making. Various barriers, drivers and practical recommendations were identified to leverage libraries as hubs to promote community health and wellbeing.

**Conclusion:**

Libraries already offer a variety of resources that directly or indirectly support the health and wellbeing of community-dwelling adults and young people. However, public awareness of these services is limited. As we navigate post-pandemic recovery, libraries can serve as platforms for community engagement, fostering resilience, mental health support and reducing social isolation. Recognising libraries’ untapped potential can lead to healthier communities and improved wellbeing.

**Supplementary Information:**

The online version contains supplementary material available at 10.1186/s12889-024-18535-5.

## Introduction

Libraries have been providing services that reflect the needs of local communities in the UK since the nineteenth century [1]. The public perception of what libraries offer may be gradually shifting because libraries are no longer used exclusively for accessing books but increasingly provide a wider range of public services that could positively impact the health and wellbeing of the community [2]. Public libraries are also considered inclusive public spaces where individuals from a wide range of ages and backgrounds can interact, acquire knowledge and exchange ideas through a variety of services, including access to the internet, formal training events and maker spaces in a community setting [2, 3].

Demand for wellbeing services has increased dramatically in recent years and since the advent of the COVID-19 pandemic [4]. Wellbeing is a broad concept essential at both the micro (personal) and macro (societal) levels and is usually measured through self-reporting [5]. It involves a holistic assessment of one’s mental health, physical health, living conditions, social life quality, ability to achieve self-potential, and overall life satisfaction [5, 6]. Improved wellbeing is associated with better health, employment, economic consequences, and social outcomes [6, 7]. For example, higher levels of wellbeing are associated with physical health benefits, including disease prevention, longevity, and even improved immune system function [5].

Publicly funded libraries offer a variety of health, educational, social, and economic services that influence wellbeing [3, 8, 9]. In addition to bridging cultural gaps as social entities with epistemic value, libraries offer a means to empower users through learning [10, 11]. They provide free and easy access to information, promote digital literacy skill improvement, support employment seeking and, in these ways, can support individuals [8, 10]. Through their role in improving universal access to information, libraries contribute to overall knowledge, digital, and health literacy, in turn increasing employment levels, skillsets, health promotion, and social involvement, all of which contribute to improved wellbeing [12, 13]. Libraries also offer social advantages to nearly all age groups [14, 15]. They provide a low-cost means for entertaining children while also improving their social and literacy skills across essential transitions in their development [14]. To the elderly or underprivileged with declining relationships and social interaction, libraries extend social, emotional and moral support and encourage connecting with similar individuals to reduce isolation [15]. As early interventionists, librarians provide self-help methods for mild-to-moderate mental health conditions in a nonclinical, embraceable, and stigma-free space [16]. Library staff also contribute to social and health equality by providing accurate, easy-to-understand guidance on health services to vulnerable groups [16]. Above all, libraries offer a sense of belonging and a level of trust to the community [17]. In a recent poll, British adults ranked librarians among the top five professions that could be trusted to give reliable information, with medical professionals topping the list [18].

The resilience and relevance of libraries have been further illuminated during the challenging COVID-19 pandemic [19–21]. Despite stringent national lockdowns altering daily life, libraries remained a beacon for many. Between 2019 and 2020, 7.6 million avid library users borrowed books, with a reported 34% of English adults visiting a library within the past year [14]. Although overall library visits have decreased, there is evidence that a large portion of the UK community still values libraries, regardless of library use [22, 23]. Despite library building closures during the lockdown, library memberships increased by 32%, 2.9 million people were proactively contacted, and more than 75% of libraries hosted online events and services [24, 25].

It is clear from the available evidence that libraries are valued for their nonclinical atmosphere and community reach [26, 27]. Health and wellbeing activities are increasingly becoming a core part of library services in the UK [28–30]. Library services are well positioned to provide health and wellbeing needs for people of different backgrounds and can therefore be ideal for helping public healthcare institutions communicate with diverse communities [21]. There is a growing literature regarding the role of libraries in health and wellbeing, rooted in earlier research, which is primarily based on case studies emphasising the most novel practices of local libraries [22, 23]. While those are informative, there is a need for a more comprehensive understanding of how public libraries can serve as essential community hubs to enhance the public health and wellbeing of community-dwelling adults. This study aims to fill this gap by exploring the potential of libraries to act as focal points for mental and physical health promotion among adults living in the community. It seeks to gather perspectives from both library users and library staff to understand better the current role of libraries in supporting community health and wellbeing and identify strategies to maximize their impact in this area.

### Study objectives

The aim of this study was to evaluate the role of libraries as community hubs. Within the context of this study, a “community hub” is defined as a central and accessible space within a community that serves as a focal point for community gatherings, resources, activities, and services, with the intent of fostering social cohesion, sharing knowledge, and promoting general well-being. The primary objective was to understand how libraries, acting as these hubs, promote public health and wellbeing among community-dwelling adults. Additionally, the study sought to assess the perceived value of libraries in current and future health promotion endeavours while identifying both barriers and facilitators for the delivery of effective wellbeing programmes.

## Methods

### Study design

**Fig. 1:**
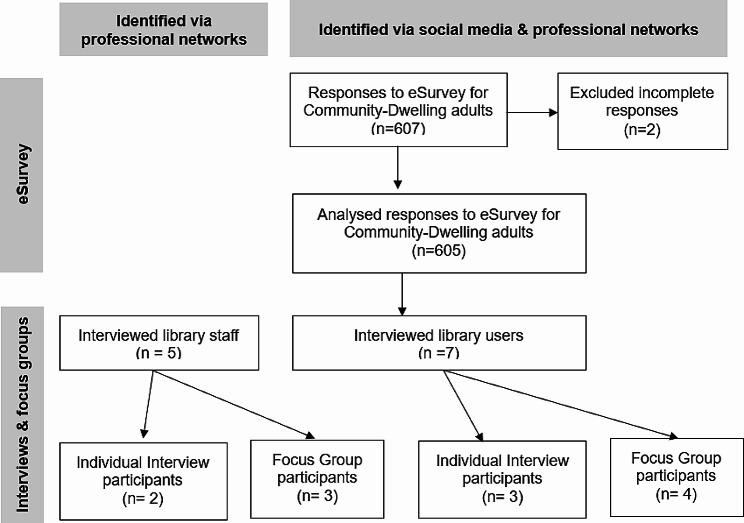
Data collection workflow diagram

A mixed-methods, cross-sectional research study (a sequential explanatory design: participant selection model) using an eSurvey and a personal interview component to explore the potential of libraries as community hubs to promote self-care, mental and physical health and wellbeing [31]. The study design involved three components [Fig. 1]. First, an online cross-sectional survey targeting community members (library users) across England. Individuals who were willing to participate in the eSurvey did so by following the link provided in the poster and social media advertisement. Following this, semi-structured interviews were conducted with seven library users, which helped us uncover themes that provided deeper insights into the data obtained from the survey. Additionally, we conducted semi-structured interviews with five library staff members to understand their perspectives on how their libraries can be repositioned to improve the health and wellbeing of their communities. A sequential explanatory design was adopted whereby contextual data from semi-structured interviews were used to interpret data collected from the eSurvey. This approach ensured that the different stages of data collection informed and enriched each other, leading to a more comprehensive understanding of the potential of public libraries in promoting community health and wellbeing.

The Health Belief model and Social Capital Theory were instrumental in shaping the eSurvey [32, 33]. The HBM is utilized to assess how individual perceptions regarding the severity of health issues and the benefits of preventive actions influence engagement with library services for health and wellbeing [33]. It examines factors like perceived risks, benefits, and barriers to health-related behaviours within the context of library usage. Social Capital Theory explores the libraries’ role in facilitating social networks, trust, and community cohesion, highlighting how these social constructs contribute to enhancing public health and wellbeing [32].

### Quantitative data (eSurvey)

#### Study population

Members of the community who were 16 years of age or older with access to a smartphone or personal computer were invited to complete a short (< 10 min) anonymous survey. Posters with a QR code and a link to the survey were disseminated across England via social media advertisement. Information about the study, including the Participant Information Sheet (PIS), was provided in the introductory section of the survey. The PIS detailed the study’s objectives, protecting participants’ data, their right to withdraw at any stage, information on where, when, and for how long data is retained, the study’s investigator, and the survey’s length. Consent for participation was obtained, and the data collected were anonymised. Survey results were stored on a secure institutional database that was only accessible by the team of researchers involved in this study. No IP addresses were collected; therefore, the team could not identify any cases of duplicate entries. Potentially eligible participants were informed that their involvement was voluntary and that it was not linked to a monetary incentive. They were informed that their participation could help advance knowledge and future policies around wellbeing services provided by libraries and that they would have the opportunity to access a summary of the research findings.

#### Data Collection

Data from 607 respondents library users were collected using an anonymised electronic survey made available on the Imperial College Qualtrics platform for three months (15 December 2021 to 15 February 2022) [Table 1]. Qualtrics websites have first-party cookies and allow third parties to place cookies on devices. The 14-item open survey was developed by a team of researchers and piloted by six researchers before its online dissemination [Table 2]. The survey included multiple-choice questions and Likert-scale responses. Questions were aimed at understanding perspectives about the importance and utility of libraries in promoting community health and wellbeing. The survey contained questions about the demographic details of the respondents, including age, gender, ethnicity, education level, and employment status, and various questions to understand their perspectives about the value of libraries in the community setting and whether the libraries are well positioned to deliver more health and wellbeing services and to help tackle inequality in health and digital literacy. Participants could review their answers before submitting them. The last question of the survey gave respondents the opportunity to provide their names and contact details in case they wished to be contacted by the study team to arrange an interview. The complete survey is available using the following link:

**Table 1:** Characteristics of Respondents

	N	(%)
**Gender**		
Female	216	(35.7)
Male	384	(63.5)
Nonbinary and Queer	5	(0.8)
**Age**		
17–24	59	(9.8)
25–34	177	(29.3)
35–44	172	(28.4)
45–54	90	(14.9)
55–64	75	(12.4)
65 and over	32	(5.3)
**Ethnicity**		
White	509	(84.1)
Mixed/multiple ethnic groups	20	(3.3)
Asian/Asian British	40	(6.6)
Black/African/Caribbean/Black British	24	(4.0)
Other (please specify)	12	(2.0)
**Education**		
Completed secondary school	79	(13.1)
Graduated from High School/College	141	(23.3)
University degree or higher	385	(63.6)
**Employment**		
Full-time employed	319	(52.7)
Part-time employed	95	(15.7)
Retired	47	(7.8)
Self-employed	46	(7.6)
Student	41	(6.8)
Unemployed	57	(9.4)

**Table 2:** Main Survey Findings

	N	(%)
**How often do you go to the library?**		
More than once a week	21	(3.5)
About once a week	59	(9.8)
About once a month	129	(21.4)
A few times a year	210	(34.8)
Rarely or never	185	(30.6)
**Which type of library do you usually go to**		
Local authority-funded library	535	(88.6)
Other (Charity run, School or University library)	111	(18.4)
**Which services are you familiar with at your local library?**		
Borrowing books or e-books or other items (e.g., CDs, DVDs, computer games, talking books)	563	(93.1)
Café/shop	140	(23.1)
Free access to Internet/computer	426	(70.4)
Photocopying/printing/faxing	383	(63.3)
Accessing information e.g., about/for the local community, Newspapers/magazines	263	(43.5)
Adult training courses on offer (literary courses, computer skills/IT sessions, employment skills, financial skills training)	117	(19.3)
Bibliotherapy activities (e.g., books on prescription, therapeutic reading groups, computer-based cognitive behavioural therapy)	34	(5.6)
Children’s activities (e.g., story time, Summer Reading Challenge)	282	(46.6)
Health services (e.g., health checks, health information & advice, exercise classes)	32	(5.3)
Lectures/readings/special events	106	(17.5)
Reading groups	121	(20.0)
Room hires	109	(18.0)
Services for groups with special needs (e.g., housebound/visually impaired)	52	(8.6)
Socialising	108	(17.9)
Space to wait/relax, work, or study	315	(52.1)
Other	12	(2.0)
**To what extent do you feel libraries are suited to promote physical & mental health & wellbeing?**		
Not at all	106	(17.6)
Slightly	72	(11.9)
Moderately	126	(20.9)
Very	185	(30.6)
Extremely	115	(19.0)
**What aspects of health & wellbeing are libraries well positioned to provide?**		
Healthy living/wellbeing centres	292	(48.8)
Library space for health checks etc.	234	(39.1)
Mental Health Awareness Days/activity	387	(64.7)
Health promotion & Health information activity	345	(57.7)
Support/provision for self-help/support	348	(58.2)
Signposting to other services	429	(71.7)
Support for other organisations (Events and activities/& use of the space)	336	(56.2)
Reading groups	448	(74.9)
Book/reading clubs with specific health or target group focus	434	(72.6)
Other (please specify)	12	(2.0)
**Visiting the library helps get me out of the house, get advice & feel connected**		
Agree	400	(66.1)
Neither agree nor disagree	127	(21.0)
Disagree	78	(12.9)
**Libraries are/can be the hub of cultural & social gatherings & activities**		
Agree	482	(79.9)
Neither agree nor disagree	63	(10.4)
Disagree	58	(9.6)
**Using the library service can help people who may be feeling isolated and/or lonely**		
Agree	533	(88.4)
Neither agree nor disagree	37	(6.1)
Disagree	33	(5.5)
**I enjoy visiting libraries to take part in clubs (e.g., books, meetings, fitness clubs)**		
Agree	195	(32.4)
Neither agree nor disagree	218	(36.3)
Disagree	188	(31.3)
**Libraries help me access digital services (e.g., internet access)**		
Agree	298	(49.4)
Neither agree nor disagree	156	(25.9)
Disagree	149	(24.7)
**Libraries are a good place for trusted information & gaining knowledge or skills**		
Agree	508	(84.2)
Neither agree nor disagree	58	(9.6)
Disagree	37	(6.1)
**Libraries help me learn about healthy eating options**		
Agree	195	(32.4)
Neither agree nor disagree	222	(36.9)
Disagree	185	(30.7)
**Visiting the library can improve my mental health & wellbeing**		
Agree	427	(70.9)
Neither agree nor disagree	130	(21.6)
Disagree	45	(7.5)
**Information about health & wellbeing services delivered by the library is not advertised enough**		
Agree	431	(71.6)
Neither agree nor disagree	126	(20.9)
Disagree	45	(7.5)
**Which of the following aspects of health & wellbeing would you like to see being promoted more often?**		
Exercise & fitness classes/activities (Taiichi, yoga for children and/elderly (e.g., with chair)	324	(53.6)
Sessions on healthy behaviours (exercise, diet, smoking, drinking etc.)	337	(55.7)
Mental health & Mindfulness activities & groups	458	(75.7)
Boardgame sessions/groups (for socialising & brain training)	344	(56.9)
Film club	215	(35.5)
Coffee morning/Chatty cafes	351	(58.0)
Knitting	159	(26.3)
A local group for people with similar health issues	235	(38.8)
Quiet place to relax or study	446	(73.7)
Friendly – spaces, staff, activities	371	(61.3)
Bibliotherapy (therapeutic reading)	172	(28.4)
Books on prescription (formal or informal recommended healthy reading lists)	186	(30.7)
E-books on health and wellbeing topics	214	(35.4)
Other (please specify)	10	(1.7)
**What do you think are the barriers to promoting health & wellbeing in libraries?**		
There is not enough funding	521	(86.1)
Library staff not being supported	353	(58.3)
Not enough awareness about services offered	411	(67.9)
Not enough public enthusiasm	260	(43.0)
Travelling to the library (maybe far or expensive)	137	(22.6)
Other (please specify)	23	(3.8)

https://imperial.eu.qualtrics.com/jfe/form/SV_2bZye8eQTDvr1NY.

#### Data analysis

Descriptive analysis was performed to summarise participants’ characteristics in frequencies and percentages. The associations between perceptions of Libraries’ Role in Promoting Physical and Mental Health and Wellbeing, and participants characteristics and frequencies of library visits were explored using Chi-square test [Table 3]. A *p*-value of less than 0.05 was considered statistically significant. All data analyses were conducted using the Statistical Package for Social Sciences (SPSS) version 28.0.1. The quality of the survey was assessed by completing the Checklist for Reporting Results of Internet E-Surveys (CHERRIES) [Additional File 1] to guide the reporting of the eSurvey [32].

**Table 3:** Association of perceptions of libraries in promoting physical and mental health and wellbeing with participant characteristics and frequency of library visits

c	Perception of libraries in promoting physical & mental health & wellbeing						
	Not at all n (%)	Slightly n (%)	Moderately n (%)	Very n (%)	Extremely n (%)	Total N (%)	*p*-value
**Age**							< 0.001
17–24	10 (9.4)	16 (22.2)	16 (12.7)	15 (8.1)	1 (0.9)	58 (9.6)	
25–34	43 (40.6)	22 (30.6)	37 (29.4)	48 (25.9)	27 (23.5)	177 (29.3)	
35–44	27 (25.5)	17 (23.6)	34 (27.0)	57 (30.8)	37 (32.2)	172 (28.5)	
45–54	13 (12.3)	8 (11.1)	15 (11.9)	40 (21.6)	14 (12.2)	90 (14.9)	
55–64	11 (10.4)	8 (11.1)	17 (13.5)	16 (8.6)	23 (20.0)	75 (12.4)	
65 and over	2 (1.9)	1 (1.4)	7 (5.6)	9 (4.9)	13 (11.3)	32 (5.3)	
**Gender**							
Male	50 (47.2)	32 (44.4)	43 (34.1)	55 (29.7)	35 (30.4)	215 (35.6)	0.025
Female	56 (52.8)	38 (52.8)	82 (65.1)	129 (69.7)	79 (68.7)	384 (63.6)	
Nonbinary and Queer	0 (0.0)	2 (2.8)	1 (0.8)	1 (0.5)	1 (0.9)	5 (0.8)	
**Ethnicity**							
White	87 (82.1)	59 (81.9)	108 (85.7)	158 (85.4)	96 (83.5)	508 (84.1)	0.977
Mixed/multiple ethnic groups	3 (2.8)	2 (2.8)	5 (4.0)	7 (3.8)	3 (2.6)	20 (3.3)	
Asian/Asian British	10 (9.4)	7 (9.7)	5 (4.0)	10 (5.4)	8 (7.0)	40 (6.6)	
Black/African/Caribbean/Black British	3 (2.8)	3v(4.2)	6 (4.8)	6 (3.2)	6 (5.2)	24 (4.0)	
Other (please specify)	3 (2.8)	1 (1.4)	2 (1.6)	4 (2.2)	2 (1.7)	12 (2.0)	
**Education**							0.702
Completed secondary school	12 (11.3)	9 (12.5)	21 (16.7)	20 (10.8)	17 (14.8)	79 (13.1)	
Graduated from High School/College	28 (26.4)	14 (19.4)	27 (21.4)	41 (22.2)	31 (27.0)	141 (23.3)	
University degree or higher	66 (62.3)	49 (68.1)	78 (61.9)	124 (67.0)	67 (58.3)	384 (63.6)	
**Employment**							0.010
Full-time employed	63 (59.4)	48 (66.7)	64 (50.8)	96 (51.9)	47 (40.9)	318 (52.6)	
Part-time employed	13 (12.3)	5 (6.9)	19 (15.1)	31 (16.8)	27 (23.5)	95 (15.7)	
Retired	7 (6.6)	2 (2.8)	12 (9.5)	13 (7.0)	13 (11.3)	47 (7.8)	
Self-employed	6 (5.7)	7 (9.7)	9 (7.1)	14 (7.6)	10 (8.7)	46 (7.6)	
Student	7 (6.6)	9 (12.5)	12 (9.5)	10 (5.4)	3 (2.6)	41 (6.8)	
Unemployed	10 (9.4)	1 (1.4)	10 (7.9)	21 (11.4)	15 (13.0)	57 (9.4)	
**Frequency of library visits**							0.057
More than once a week	2 (1.9)	2 (2.8)	4 (3.2)	6 (3.2)	7 (6.1)	21 (3.5)	
About once a week	7 (6.6)	3 (4.2)	17 (13.5)	20 (10.8)	11 (9.6)	58 (9.6)	
About once a month	18 (17.0)	14 (19.4)	24 (19.0)	42 (22.7)	31 (27.2)	129 (21.4)	
A few times a year	34 (32.1)	28 (38.9)	38 (30.2)	66 (35.7)	44 (38.6)	210 (34.8)	
Rarely or never	45 (42.5)	25 (34.7)	43 (34.1)	51 (27.6)	21 (18.4)	185 (30.7)	

### Qualitative data (interviews)

#### Study population

The qualitative phase of the study encompassed interviews with a convenience sample comprising both library users and library staff. After completing the survey, Library users were given the option to indicate their willingness to be contacted for an interview. Personal and professional contacts were leveraged to identify potential interviewees among library staff. Library staff interested in participating in the interviews responded to the email invitation, expressing their interest and seeking further details about the study.

All participants who expressed their intention to participate in the interviews were contacted via email. This email invite included the PIS and an electronic consent form link. The perspectives of the community members and library staff were gathered from those participants who granted their consent to be contacted for interviews.

#### Data collection

A total of 12 interviews comprising seven library users and five library staff members (public-facing staff or senior members of management) were conducted. The interviews lasted between 25 and 60 min and were informed by interview guides and allowed saturation. Insights from the preliminary survey phase, guided by the Health Belief Model and Social Capital Theory, further informed the interview guide development and served to further explore participant survey views.

Three researchers not previously known to participants conducted the interviews virtually using Microsoft Teams. The interviews were audio-recorded, auto-transcribed, and subsequently checked manually for accuracy. Only three authors, AEO, MK and MA, interviewed participants and had access to complete transcriptions of interviews. Neither the interviews nor the transcripts were repeated or returned to participants. Participants were informed that any ad verbatim quotations used to highlight central themes would be made anonymous.

#### Data analysis

MK, female, carried out the data analysis with support from MA and ERS (both female). The interviews were reviewed multiple times and underwent thorough analysis to ensure comprehensive data capture and saturation. Detailed thematic analysis was conducted on the interview notes, following a systematic process involving familiarisation, initial code generation, theme identification, theme review, and theme definition and naming [Table 4]. Researchers reviewed the transcripts and agreed on emerging themes, which were later discussed with a wider research team. Emerging themes were grouped into clusters and categorised. Anonymised and verbatim quotes from the transcripts were noted to illustrate a selection of key themes [Additional File 3]. The Consolidated Criteria for Reporting Qualitative Research (COREQ) [Additional File 2]. A checklist was used to guide the reporting of interviews [33]. The study team did not discuss findings with participants but was keen to share publications with anyone who expressed interest.

**Table 4:** Thematic analysis of respondents’ views on barriers and drivers to the promotion of health and wellbeing in public libraries

Drivers to the promotion of health & wellbeing	Positive perception of libraries	“Safe place”, quiet, friendly & familiar, welcoming to diverse people
		Social space, encounter new people, spend quality time with family
		Resourceful space, reliable source of information, borrow books, learn
	Positive evolutions	Shift to digitisation
		Emphasis on community hubs
		Diversification of services offered
	Diversity of existing services	Information stalls for health awareness
		Collaboration on various health topics
		Yoga, meditation, mindfulness
		Coffee mornings, ESOL groups
		Art, dance, and educational courses
		Law & police contact points
Barriers to promoting health & wellbeing	Lack of community awareness & campaigns	Users unaware of existing health & wellbeing resources & activities
	Funding concerns	Service limitations, high expectations with limited resources
	Staff-related barriers	Lack of involvement, training, and communication, staff feeling devalued, lack of staff
	Outdated & fragmented services	Not youth-centred, perceived as uncool, fragmented service provision
Recommendations to better promote health & wellbeing	Increasing awareness & targeted interventions	Activity scheduling, technology outreach, special sessions for isolated individuals
	Diversified & accessible service provision	Expand online services, strategies for awareness & youth engagement
	Hiring specialised staff	Dedicated staff for health & wellbeing, better decision-making involvement, needs assessment, staff training

### Ethics

The study was given ethical approval by the Imperial College Research Ethics Committee (ICREC #21IC7274 on 03/12/2021).

## Results

The survey gathered responses from 607 individuals; however, after excluding incomplete responses, a total of 605 library users’ data were analysed. Contextual interviews were conducted with 12 participants, consisting of seven community members and five library staff.

### Demographic profile of study participants: eSurvey

Out of the 605 library users who participated in the electronic survey, males represented a substantial portion (63.5%) of the cohort compared to female (35.7%) participants. More than half (57.7%) of the respondents were aged between 25 and 44, and the White demographic was overwhelmingly predominant (84.1%). In terms of educational background, more than half (63.6%) had a university degree or higher, and 58.4% were in full- or part-time employment. The characteristics of the eSurvey respondents are shown in Table 1.

### Survey findings

Over half of the respondents (65.4%) rarely visited the library/only a few times a year, compared to 13.3% who frequented the library more than once a week [Table 2]. A significant majority (88.6%) frequented local authority-funded libraries, as opposed to charity-run, school and university libraries (18.4%).

Most respondents were familiar with traditional library services such as borrowing books (93.1%), free access to a computer/internet (70.4%), and using a photocopier, printer, or fax machine (63.3%). A little over half (52.1%) use libraries as public spaces to wait, relax, work, or study. However, significantly fewer respondents were familiar with some of the more specific health and wellbeing-related services, such as health checks, health information and advice and exercise classes (5.3%), bibliotherapy (5.6%), socialising (17.9%), or services for groups with special needs (8.6%), including activities for individuals who are housebound or have a visual impairment.

Almost half of the respondents (49.6%) felt libraries are ‘very’ or ‘extremely’ suited to promote overall physical and mental health and wellbeing in service users. In contrast, 17.6% felt that libraries are not at all suited for this role. A vast majority (84.2%) agreed that libraries are good places to acquire trusted information, new knowledge, or skills, and 66.1% believed that visiting the library helps them get out of the house, get advice, and feel connected. Additionally, a significant 79.9% (482) believed that libraries can be the hub of cultural and social gatherings. Many respondents (88.4%) also agreed that library services could be beneficial for those feeling isolated or lonely, improve their mental health and wellbeing (75.5%) and that libraries can be a hub of cultural and social gatherings and activities (83.3%). When queried about potential health and wellbeing services they would like to see more in libraries, 75.7% of respondents expressed interest in libraries promoting mental health and mindfulness activities and groups, while 73.7% would appreciate a quiet place to relax or study.

A significant 71.6% of respondents believe that health and wellbeing services provided by libraries are not advertised enough. When asked about barriers to promoting health and wellbeing services in libraries, the most common barriers identified were ‘lack of funding’ (86.1%), ‘lack of awareness about services offered’ (67.9%), and ‘library staff not feeling supported’ (58.3%).

Analysing the association between perceptions of libraries in promoting physical and mental health and wellbeing and participants characteristics and frequency of library visits [Table 3]. Age displayed a significant variation in perceptions, with the 35–44 age group primarily believing libraries are ‘Very’ suited for wellbeing promotion (*p* < 0.001). While both males and females have a similar trend, gender differences are also notable, as a higher proportion of females believe libraries to be ‘Extremely’ suited for the purpose compared to males (*p* = 0.03). Lastly, employment status showcased an association with full-time employed individuals predominantly viewing libraries as ‘Very’ suited to promote wellbeing (*p* = 0.01). While the association between the frequency of library visits and the perception of their role in wellbeing is on the verge of statistical significance (*p*-value = 0.06), some notable observations include: people who visit the library a few times a year or rarely or never have a more varied perception, with responses spread across ‘Very,’ ‘Moderately,’ and ‘Rarely or Never.’ A significant portion of those who visit approximately once a month (27.2%) perceive libraries as ‘Extremely’ suited for promoting health and wellbeing. Overall, findings suggest that people’s perceptions of the role of libraries in promoting health and wellbeing are influenced by factors like age, gender and employment status. However, factors like ethnicity, education level, and frequency of library visits might not play a significant role in shaping these perceptions.

### Interviews

#### Demographic profile of interview participants

Two focus groups and six interviews, ranging from 25 to 60 min each, were conducted with a total of 12 participants (five library staff and seven library users) during the study timeline (Table 5). The library staff sample included library managers and community engagement officers; three were female, and two were male, ranging between 35 and 50 years old. The library user sample consisted of four females and three males aged between 19 and 45 years old.

**Table 5:** Characteristics of Interviewees

-	N	(%)
**Total**	12	(100)
**Gender**		
Female	7	(58.3)
Male	5	(41.7)
**Age**		
19–30	5	(41.7)
30–40	3	(25)
41–50	4	(33.3)
**Ethnicity**		
White	5	(41.7)
Black/African/Caribbean/Black British/other	6	(50.0)
Mixed/multiple ethnic groups	1	(8.3)
**Designation**		
Library Staff	5	(41.7)
Library user	7	(58.3)

A total of 10 themes were identified from the interviews and classified as (1) Drivers, (2) Barriers or (3) Recommendations. [Table 4].

#### Drivers to the promotion of health and wellbeing

- **Positive perception of libraries**.

When asked about their perceptions of libraries, library users and staff felt that libraries, as public buildings, are considered a ‘safe place’ by many and that they are often “a quiet and productive place”. Almost all participants felt that the libraries were friendly, familiar, and welcoming to people from all walks of life. There was also unanimous agreement that libraries offered a good range of activities for various groups with different needs.

“It was somewhere familiar and safe and welcoming and a place I could go to. There are sessions like coffee mornings, where local elderly people could go and sit and chat, and a regular member of staff would coordinate it so they had that familiarity.” (Library users: P3, age 39).

“…people were walking into libraries as soon as we opened after the lockdown. It is like they gravitated to libraries. That is because we’re free, we’re safe, we’re non-judgmental. Even now, people complain that they’re trying to get hold of other departments in the council, but the library - we’re frontline. Our doors are always open, and we do not have barriers like saying you are not welcome.” (Library staff: P10, age 45).

Members of the community felt that libraries were not only a reliable source of information but also a place to borrow books and learn new things. For some, the library had sentimental value, such as recollecting memories and passing on the love of books and libraries to children.

“You know I will probably never stop going to the library. You know I have passed that [tradition] on to my daughter in the same way that it was passed on from my parents, so I hope my daughter will keep going.” (Library users: P11, age 37).

Almost all respondents considered the library as a social space where there is routine interaction between people, and the interactions were different in that “it is not like we’re buying and selling things.” A library-using mother felt the library was a space to spend quality time with her children. Two library users interviewed did not perceive the library as a place to socialise and connect with others.

“To be honest, no! Like I do not go to a library to socialise, and I feel like when I go there, I go there to be productive, and for that, I need to be quiet. I do not see the library as a place where I can hang out with my friends. Maybe it will change.” (Library users: P7, age 19).

(b) **Positive evolution of libraries**.

Almost all staff acknowledged that over time, libraries have undergone substantial transformation in management, operations, and service offerings. Positive changes included the diversification of services that can help build community resilience and improve literacy levels. The role of library staff has also changed to include more community and person-centred approaches.

“…When we interview for a job at the library, we’re really looking for someone who loves people because it is not just about borrowing books and CDs now. It is about interacting with people and being at the heart of the community.” (Library staff: P1, age 50).

(c) **Existing health and wellbeing services**.

When asked what existing health and wellbeing services or activities were available in their library, all librarians were aware of the services and listed various activities provided by their library, particularly services related to health and wellbeing.

“People often think that library is just about education. Rightly, libraries are about reading for pleasure as well. We have online resources. We have online courses to help people benefit, better themselves, education-wise we have got, I think, Open University courses. We have got all different things that are available to people. I do not think they realise that we do that half the time.” (Library staff: P6, age 42).

These services included free yoga and meditation sessions as well as punctual collaboration on health-related topics. In addition, librarians indicated that social and community services such as online coffee mornings, ESOL (English for Speakers of Other Languages) conversation groups, art, dance, and educational courses have been particularly popular among users. Finally, libraries can also serve as law and police contact points.

#### Barriers to promoting health and wellbeing

Lack of community awareness, funding concerns, staff-related barriers, and services were identified as barriers to promoting health and wellbeing in the community.

- **Lack of community awareness**.

All participants agreed that community members are unaware of all services provided by the library. Very few library users knew that libraries offer such health and wellbeing services or, at most, were only able to list a few available services.

“I have not heard. I have not been aware that libraries do that kind of stuff, and I think that would be a great idea, especially if it was free. However, no. Yeah, I had no idea that you could get that. I think if I knew that there were that sort of like activities or services available, I would probably at least consider popping along.” (Library users: P12, age 27).

In response to a question about library promotion and marketing, librarians stated that using “word-of-mouth” and “making people aware of the activities when they visit the library” were common practices used to spread awareness of health and wellbeing services. After the COVID-19 pandemic, librarians used social media for the first time to promote their services and connect with their communities.

“Some people do not ever come into a library, so would not know. Some people do not use social media, so would not know. Therefore, if those two things are combined, how would they know about the library service? Therefore, what one thing we need working on. “(Library staff: P6, age 42).

“They are like, yeah, I’m sure that they are a bit of an untapped resource like we do not have people knowing, yeah, but it is available.” (Library users: P11, age 37).

(b) **Funding concerns**.

The librarians felt there was a limitation to what could be offered due to high demands on them without proper funding, staffing, and training. In parallel, most library users acknowledged that services are extremely stretched, budgets are tight and that lack of funding could be linked to issues with staffing and services.

“You know, we are the only council’s walk-in service. Anyone can come in, there’s no restriction and because of all the cutbacks, there are hardly any community centres or day centres, so we are really important. But with that comes quite a lot of issues as well that we’re not always very well equipped to deal with because we get a lot of.” (Library staff: P4, age 35).

(c) **Staff-related barriers**.

Most library-using participants mentioned that library staff were amiable, helpful, warm, and welcoming. Many users mentioned that staff had gone above and beyond to help people in need, and when they could not, they pointed users in the right direction to get further help. On the other hand, many participating librarians felt devalued. Some staff cited the hurdles to promoting health and wellbeing when using libraries as a vessel, such as lack of staff dedicated to specific aspects of library activities, lack of training and involvement in decision-making. Additionally, resistance to change could be felt from some staff participants during the interviews.

“I mean, it is fascinating, isn’t it? Because it boils down to a very specific collection of skills that are quite difficult to acquire. Liberians are sort of counsellors, advisors, performers, straddlers and critics. Yeah, you know, it is like a dilettante sort of sharing your appreciation of things. It is such a range of skills, and yet the staff are generally, I think, are quite devalued.” (Library Staff: P5, age 49).

(d) **Outdated services**.

In addition to being unaware of the services provided, some librarians felt that services and activities on offer were “not connected to today’s youth”. Few young library-using participants mentioned that there is a potential for libraries to be perceived as “uncool” and that young people may avoid libraries due to fear of bullying. Other concerns raised by library users were inadequate access to services, a faulty perception of the library, a lack of designated areas for activities, timing for activities that were often inconvenient, and challenging user behaviour.

“Also, I feel like there’s a stereotypical library image where younger kids are looked down upon. So, like going to a library, you may be considered a geek or stereotypical stuff like that, but that needs to change because children, especially mental health is becoming increasingly aware we need places where kids can actually go and become educated and have a safe place to go.” (Library users: P7, age 19).

“Young people from local schools asked her if they could use the office after school because they wanted to do some schoolwork and so on, but they did not want to do it in the library there. They wanted to come to her office.” (Library Staff: P5, age 49).

#### Recommendations

When asked what needs to change in libraries so that they are better equipped to promote public health and wellbeing successfully, key recommendations included increasing community awareness, providing diversified and accessible services, and hiring specialised staff.

- **Increasing community awareness**.

All participants mentioned that increasing awareness about the breadth of services provided by local libraries is key. Service users felt that building community trust and assessing and addressing the community’s needs where possible were important starting points. Many stated that targeted communication and promotion strategies tailored to different segments of the community are imperative to connecting with youth or people who may be digitally excluded.

“I think, you know, the library could just do that little bit more to sort of push itself into the community and into schools, and since they’re doing so many lovely things with children and with adults, I think they could be a bit more confident about what they have on offer and push it out there because I think they have got people like me hooked.” (Library users: P10, age 45).

(b) **Diversified and accessible service provision**.

Several library users and library staff felt that continuing and increasing their online activities and services, which were held during the pandemic, would be beneficial. They also pointed out that balancing in-person and online services is vital even with increasing digitisation and to ensure equal access for members. A few library users also felt that there should be targeted activities and services aimed at catering to a different audience at different times, such as morning and afternoon activities designed for older people or people who have been quite isolated throughout the COVID-19 pandemic and mothers with young children. More respondents proposed increasing the provision of afterschool activities for youths and office workers in the late afternoon to evening period. Many participants stressed the need for separate dedicated spaces to host activities so that they do not encroach on quiet spaces.

“You know, it is about getting the balance right, and I think, as we’re all aware of, the need to be with other people physically is almost greater than the need for, you know, just being able to have that flexibility and to do something from the comfort of your own home. Actually, you do need to meet people and interact.” (Library users: P11, age 37).

(c) **Hiring specialised staff**.

Most staff members indicated that hiring individuals dedicated to promoting health and wellbeing would help further promote community engagement and resilience. All respondents felt that involving staff in decision-making and providing more funding and training to support library users better could help promote health and wellbeing.

“I think I would want to have dedicated staff employed to start some of these projects, and it shows because, at the moment it is very haphazard and you know, if you had a dedicated person or two people, then you would have the time and the energy to really put into and you know use the library in that way.” (Library staff: P2, age 49).

## Discussion

### Main findings

This study sought to evaluate the viability of libraries as catalysts in boosting community health and wellbeing. The results indicate that libraries can indeed function as secure, trustworthy sources for health and wellness information if existing barriers are adequately addressed. Both library staff and service users expressed similar viewpoints on how libraries can be repositioned to improve their planning and delivery of health and wellbeing services. Overall, the findings suggest that there is a great public interest in utilising health and wellbeing services, provided that proper awareness is extended to the community through diversified and targeted communication and that additional support is granted to librarians and other library staff members.

### Comparison with existing literature

Consistent with prior studies, fostering a community sense of belonging can significantly elevate overall wellbeing [34]. While individual factors undeniably influence wellbeing, external factors such as community connectivity and local pride also play pivotal roles, as highlighted in policies such as the ‘Wellbeing of Future Generations (Wales) Act 2015’ and ‘The UK Government’s Levelling Up Policy’ [35–37]. This study also revealed that libraries’ potential as health and wellness pillars is augmented by the high trust and support they receive from their users. These findings are consistent with previous survey results in the UK, Canada and the US, where the public agreed that libraries are safe places and trusted sources of information, highlighting the position of libraries as welcoming hubs for community action and wellbeing [18, 38–40]. Various successful initiatives across the UK highlighted the vital role that libraries can play in helping to tackle “wicked problems”, including social isolation, loneliness, and poor mental health [41–43]. The consistent support for libraries regardless of how often individuals used the service also aligns with the findings from a previous online survey conducted in the UK in 2015, which indicated that 90% of respondents believed their library service should be preserved, regardless of whether they used the service regularly or not [44, 45].

The literature also supports the involvement of staff in decision-making and early input as. a way to help them feel supported and valued, positively affecting service delivery, implementation, and outcomes [46]. Increasingly, during tough financial circumstances, the library staff’s in-depth knowledge of users can add high value to service design, planning, and delivery [46]. With local authorities implementing a reduction in funding, libraries may benefit from saving costs and diversifying their streams of income to support and develop new and existing health and wellbeing services such as fundraising, partnering with the private sector, and exploring opportunities to partner with other libraries through the integration of shared resources and services [46, 47].

### Policy, economic and research implications

Evidence suggests that a rigorous and disciplined marketing and promotion approach has been shown to improve the outreach of library resources to match community needs for optimal impact [46, 48]. Such systematic approaches help improve the visibility of the library to promote the hidden value of public libraries to community members [49]. The Library Taskforce has developed a toolkit to encourage libraries to identify local area needs, measure the impact of local library services, and redesign and improve services to develop more evidence-based provisions [46, 50]. For instance, initiatives such as ‘Read my Mind’ by the Norfolk Library, which gained popularity after the COVID-19 pandemic, have allowed for a wider community outreach programme through online platforms to tackle the negative issues of mental wellbeing [51]. Despite the concern of digital exclusion when engaging with the public through online activities, a recent independent evaluation of ‘Engaging Libraries Phase 2’ showed that participants had greater confidence, active participation, convenience, and relevance to those shielding and unable to leave their home due to the COVID-19 lockdown [21, 52]. Participants expressed positive comments about online activities run by libraries and the desire for these online activities to continue alongside face-to-face activities after the COVID-19 lockdown [20, 53]. Although promising, the shift towards digital modalities must be balanced with efforts to bolster digital literacy, offering comprehensive training and support.

The educational, social, and cultural value of libraries and their positive influence on the community’s mental and physical wellbeing is evident [19]. However, measuring the potential long-term direct and indirect economic contribution/monetary valuation of libraries and their services/activities is challenging but essential [8, 40]. A cost‒benefit analysis (CBA) methodology pioneered by The Greater Manchester Combined Authority (GMCA) Research Team has been used by public, private, voluntary and community sector partners across the country to consider ‘the value for money’ presented by different interventions that may otherwise not be straightforward to compare [54]. The CBA model can enable libraries to make a more comprehensive ‘economic case’ or for public value to be articulated fully, enumerating the economic and social benefits that could reflect on individuals and businesses in terms of improved individual health and wellbeing and as an output quantification of the return-on-investment potential. The study also highlights the importance of raising community awareness. It sheds light on barriers to efficiently promoting health and wellbeing in library settings as perceived by participants, which could potentially influence policymaking and future funding opportunities lended to libraries to mitigate these challenges. Overall, wellbeing is a multifaceted entity influenced by factors ranging from an individual’s quality of relationships, health, places they call home, income, and other interlinked factors. The influence of each on wellbeing cannot be understood in isolation and with declining gross domestic wellbeing (GDWe) against the backdrop of the COVID-19 pandemic, which has further exacerbated and exposed social and economic inequality [55–58].

Libraries have the potential to act as a buffer zone for community members, policymakers, governing bodies, and organisations to come together to promote and simultaneously mitigate hurdles to the promotion of health and wellbeing in the community setting [59]. Further research is essential to holistically understand the intricacies of this dynamic and determine the best pathways forward. Attention should be given to the perspectives of Black, Asian and Minority Ethnic (BAME) individuals as Black and Asian ethnic groups (49.3%) showed higher public library use in England than white ethnic groups (31.8%) according to the latest Taking Part survey [60]. In order to avoid reinforcing various forms of inequality, whether economic, cultural or digital, policymakers must take account of the existence of different groups of users with specific needs, preferences and practices [61].

### Strengths and limitations

To our knowledge, no previous studies in the UK have explored this specific topic (the potential of libraries as community hubs to promote public mental health and wellbeing in the general population), considering the views of both library users and librarians. One strength lies in the novelty of the study’s focus, contributing unique insights into a topic that has gained renewed importance in the wake of the pandemic. The qualitative approach adopted in this study allowed for an in-depth exploration of participants’ perceptions and experiences, providing rich and nuanced data. A clear strength lies in its diverse age representation, providing perspectives from young adults to senior citizens, which enriches the age-related understanding. Additionally, the broad spectrum of socioeconomic perspectives gleaned from the varied employment statuses of participants enhances the study’s depth.

While the study captures diverse demographics, it is predominantly represented by males, individuals aged 25–44, and those of white ethnicity. Furthermore, a significant majority of participants had a university degree or higher. These skewed representations might limit the study’s comprehensiveness and the generalizability of its findings. The underrepresentation of certain age and ethnic groups means that the study might not fully capture the perceptions unique to these demographics. Additionally, the voluntary nature of participation in both the eSurvey and interviews introduces the possibility of self-selection bias, whereby respondents inclined towards library usage might be overrepresented, potentially affecting the representativeness of the UK population. In light of the incidence-prevalence bias, also known as Neyman bias [62], it is crucial to acknowledge the potential for participants already engaged with library services to be more likely to participate, potentially leading to an overestimation of libraries’ impact on health and wellbeing within the broader population. Future research endeavours could consider strategies to address this bias and enhance the study’s validity.

## Conclusion

In conclusion, this study highlights the latent and often overlooked potential of libraries as invaluable resources for enhancing community health and wellbeing. Libraries, equipped with a diverse array of resources, hold the capacity to directly or indirectly contribute to the betterment of health and wellbeing across diverse demographics, encompassing everyone from community-dwelling adults to youth. However, to maximise this potential, it is imperative to address the existing disconnect between libraries and the communities they aim to serve. There is a pressing need to not only promote what libraries offer but also to ensure that these offerings align with the evolving needs of the community. This necessitates strategic community outreach campaigns that effectively communicate the available resources and services. Furthermore, securing consistent funding and investing in professional development for library staff is critical [63]. Engaging staff in decision-making processes instils a sense of ownership and ensures that services are tailored to meet community needs.

In the wake of the COVID-19 pandemic, as we transition towards a new normal, libraries emerge as crucial epicentres for community cohesion and engagement. They can act as bulwarks, strengthening community resilience and supplementing public mental health initiatives, particularly in combatting issues such as loneliness and social isolation. More than just repositories of books, libraries can serve as beacons of hope and connection, helping to bridge societal divides [63]. Embracing the multifaceted role of libraries can lead to healthier, more interconnected communities, setting the stage for a more promising and harmonious future.

### Electronic supplementary material

Below is the link to the electronic supplementary material.

Supplementary Material 1

Supplementary Material 2

Supplementary Material 3

Supplementary Material 4

## Data Availability

The data that support the findings of this study are available from the corresponding author, MK, upon reasonable request.

## Declarations

### Patient and public involvement

Patients were not involved.
