# Supplementary material for: Public libraries to promote public health and wellbeing: a cross-sectional study of community-dwelling adults
Source: BMC Public Health. 2024 May 3;24:1226. doi: 10.1186/s12889-024-18535-5 (PMC11069228; doi:10.1186/s12889-024-18535-5)
Supplement: Supplementary file 3 — Supplementary Material 3 [file 12889_2024_18535_MOESM3_ESM.pdf]

Additional File 3: Thematic analysis of respondents' views on barriers and drivers to the promotion of health and wellbeing in public libraries (with supporting quotes)

**Drivers to the promotion of health & wellbeing**	Positive perception of libraries	• "Sessions like coffee mornings, where the local elderly people could go and sit and chat, and it would be coordinated by a regular member of staff so, they had that familiarity and it was somewhere familiar and safe and welcoming for them to go up, you know."<br>• "I would say keep doing all the brilliant work that you're doing, even though you know you're constantly having your resources and your funding stripped back. Just please please, please keep doing and keep doing more of what you're doing because you know you were a great asset to the community and if we lose you, we will not get you back."<br>• "Especially during lockdown, a lot of the aspects of the Council shut down and you know people were looking to libraries as soon as we open, people gravitated to libraries. We're free, we're safe, we're nonjudgmental. Even now people are complaining that they're trying to get hold of other departments in the Council, and nobody is responding. But the library, we're frontline, where the doors, are always open. We do not have barriers to say you are not welcome."<br>• "I did not have a lot of time to myself to being a full-time mom. You know, to look for things just for me, but you know when it was available, I did access it and you know they put a lot of material on digital use, so we access that which was really helpful."<br>• "You know I will never probably stop going to the library. You know I have passed that on to my daughter in the same way that it was passed on from my parents so my daughter will keep going."<br>• "And we're sort of one of the remaining safety net if they have fallen through other all the other safety Nets, we are sort of the last."

	Positive evolutions	"Nowadays, if you go for a job interview at the library, what they're looking for, is someone who's, you know, loves people because it is not just about books about borrowing books Now, it is about interacting with people and, you know, being at the heart of the community." "Will not say it will be a decrease in physical book borrowing, but I feel like more like digital versions of books. All going to be shifted online and become more digital because even now a lot of people access their books online and there are services and activities online, especially after COVID." "Right now, presently some libraries, have spaces where you can speak. I'm seeing more like compared to like 2013, 2014 I'm seeing like places where you can socialise. So it has been increasingly getting better."
	Diversity of existing services	"People often think that library is just about education. Rightly, libraries are about reading for pleasure as well. We have online resources, we have online courses to help people benefit, better themselves, education wise we have got, I think, Open University courses. We have got all different things that are available to people. I do not think they realise that we do that half the time." "You know, like the Home Life service. We have a home library service which goes out into the community. So really, they're ticking the box on some of those aspects. For people who are housebound and cannot get into the library." "I mean it, it is a nice list, you know. I mean, right on unhealthy eating. Remember the goal born 5 a day, healthy hearts that sort of thing. Yeah, blood pressure tests as well."

**Barriers to promoting health & wellbeing**	Lack of community awareness & campaigns	• "The barriers for some people do not ever come into a library so would not know. Some people do not use social media so would not know. So, if those two things are combined, how would they know about the library service? So that one thing we need working on."<br>• "There's no sort of publicity to get people involved."
	Funding concerns	• "In addition to the core library services – health and well-being related services come and go depending on the funding, staff."<br>• "Uh, so there's lots that could be done, but it is having the time, staff, resources, and support and partnership working too"
	Staff-related barriers	• "You know, we are the only Councils walking service. Anyone can come in, there's no restriction. And because of all the cutbacks, there are hardly any community centers or day centers, so we are really important. But with that comes quite a lot of issues as well that we're not always very well equipped to deal with because we get a lot of."<br>• "..because I think at the end of the day we have to also be clear. Even though like we reliably and we, we can be everything. We are a library at the end of the day. We are not trained to be healthcare professionals, counsellors, or social workers. You know our business is books learning supporting. I do not think we should move too much into the arena as we are not a day center."<br>• "You know it takes time to do these and resources and I do not feel that we often have that amount of time to really dedicate.<br>• "On the one hand libraries are being sort of imagined to be places where skilled, sort of community development. Yet, on the other hand, people feel quite a sort of D-professionals and D skills, and they refer to as assistants."

		• “I mean, it is fascinating, is not it? God, because it is, it boils down on the ground to a very specific collection of skills that are quite difficult to acquire. Liberians as sort of counselors/advisors/performers straddle/critics. Yeah, you know, it is like dilettantes sort of sharing your appreciation of things. It is such a range of skills and yet the staff are generally, I think for your quiet devalued”
	Outdated & fragmented services	• “….because we're trying to do our job and then pick up bits and pieces of other initiatives. UM, and that is quite frustrating. 'cause then you feel you have not, You know you're you're running touching on what needs to be done? Yeah, in an ideal world we would do a lot more, could do a lot more with the right support and partnership working.”<br>• "To be honest, no. Like I do not go to a library to socialise, and I feel like when I go to the library, I go there to be productive or be quiet. I do not see the library as a place where I can hang out with my friends. Maybe it will change."<br>• "Also, I feel like there’s a stereotypical library image where younger kids are looked down upon. So like going to a library and you may be considered like a geek or stereotypical stuff like that, but that needs to change because children's or especially mental health is increasingly becoming an issue. we needed places where kids can actually go and become educated and have a safe place to go."<br>• “Young people from local schools asked her if they could use the office after school because they wanted to do some schoolwork and so on but they did not want to do it in the library there. They wanted to come to her office.”
	Increasing awareness & targeted interventions	• "I think you know, the library could just do that little bit more to sort of push itself into the community, into schools and since they're doing so many lovely things with children and with

**Recommendations to better promote health & wellbeing**		adults, so I think they could be a bit more confident about what they have on offer and push it out there 'cause I think they've got people like me hooked."<br>• "I have not heard, I have not been aware that libraries do that kind of stuff and I think that would be a great idea if especially if it was free. But no. Yeah, I had no idea that you could get that. I think if I knew that there were that sort of like activities or services available, I would probably at least consider popping along."
	Diversified & accessible service provision	• "Young people just drifting into, probably a life of mischief, can we help with that? what can we offer that will deter them from that, and guide them, give them training opportunities. You know, having presentations, having inspirational speakers in our space once a month or something like that, we're trying to think of different ways we can connect with people so that people within our community see us."<br>• " it is in the public space, it does not cost them anything to do it if you know if it is there and then if they want to hire A private room, then that is something different like you know, because there are not these community hub spaces anywhere, that is where libraries are best placed to be able to do it."<br>• "You know it is it is getting the balance right and I think as we're all aware of the need to physically be with other people is almost greater than the need for, you know, just being able to have that flexibility and to do something from the comfort of your own home. Actually, you know, you do need to meet people and interact."<br>• "… yeah, we have got this, you know, the world is changed by doing things virtually but really is it the best way and is it the best way to target people who maybe are more vulnerable to health and well-being issues? I'm not 100% sure, but maybe it is a way to get people in first of all and then encourage them to do something and face to face. Perhaps that is the way forward."

	Hiring specialised staff	• “I think I would want to have dedicated staff employed to do, you know, start some of these projects and it shows in because at the moment it is very haphazard and you know if you had a dedicated person or two people then you would have the time and the energy to really put into and you know use the library in that way”<br>• “I imagine in terms of, like homelessness or people with addictions like, there would have to be a lot more investment in training and providing staff. I guess equipping them to be able to do that …. so I guess if they had the right kind of help to be able to do that. I'm sure that could happen.”
