# Supplementary material for: Public libraries to promote public health and wellbeing: a cross-sectional study of community-dwelling adults
Source: BMC Public Health. 2024 May 3;24:1226. doi: 10.1186/s12889-024-18535-5 (PMC11069228; doi:10.1186/s12889-024-18535-5)
Supplement: Supplementary file 4 — Supplementary Material 4 [file 12889_2024_18535_MOESM4_ESM.pdf]

Interview guide

Library User

General Questions

1. When you hear the word "libraries" what springs to mind?
2. In your opinion, how have libraries changed in the last decade?
3. Is it a good change or bad? / What are your thoughts on the diverse role libraries play these days?
4. Have you used any public library recently? And for what reason?
5. Are you aware of services provided by your local library? Would you kindly list some for us?

Health & wellbeing specific questions

6. Have you used a public library for any health or wellbeing-related reason, for example, borrowing a book about a health matter, taking part in a reading group (mental health), or getting information about local groups? If yes, please describe your experience. If No, please tell us why not.
7. Where did you hear about the service and activities?
8. Do you know what health and wellbeing services/ activities are currently offered in your library? Please provide a brief description.
9. What does your library do to promote wellbeing?
10. How can libraries reposition to promote health and wellbeing in the future?
11. Do you see this as something that libraries would be good at? Why?
12. What would encourage you to access a library more often? Specifically, to access health and wellbeing services?
13. What health and wellbeing services or activities would you be interested in accessing at a library?
14. What are your views on the potential for an all-England health and wellbeing offer for libraries, and what would it look like if one was developed?
15. Do you think libraries have the potential to tackle wicked problems such as loneliness, homelessness, alcohol, or addiction? If so, how?

Library Staff

General Questions

1. In your opinion, what has changed about libraries in the last decade? Is it a good change or bad? What is your thought on the diverse role libraries plays these days?
2. Would you please describe your more frequent library service users?
3. What services/ program of the library is most utilised?
4. What sort of service do you provide in general?

5. Who are your partners? / What organisation, association's, institutions, and local groups (faith-based org, arts org etc) is your library connected to?

Health and wellbeing specific questions

6. Are there any health and wellbeing services activities offered in your library currently? Please provide a brief description
7. What percentage of library users use the health and wellbeing services on offer? Are you able to track usage of wellbeing services?
8. How do you generate ideas for health and wellbeing services you can offer at your library?
9. What has/is worked/working well when it comes to improving health and wellbeing services?
10. What promotion and marketing techniques are used for the health and wellbeing services and events offered, and what is most effective?
11. Please list all the partners the library service works with on health and wellbeing projects.
12. The library should reflect the cultures of the community around it while considering health and wellbeing services. What is your thought on this?
13. What are the barriers/challenges that you are facing while promoting health and wellbeing?
14. Do you receive regular feedback regarding your health and wellbeing activities? How do you reinforce positive feedback and address the negative ones?
15. What do, in your opinion, need to change the future for libraries to be promoting health and wellbeing?
16. Are there any training programs in place to equip library staff in this sector?
17. What are your views on the potential for an all-England health and wellbeing offer for libraries, and what would it look like if one was developed?
18. Do you think libraries have potential to tackle wicked problems such as loneliness, homelessness, alcohol, or addiction? If so, how?
19. Has your library previously organised event to tackle wicked problems? Can you list some?
